# Characterisation and Germline Transmission of Cultured Avian Primordial Germ Cells

**DOI:** 10.1371/journal.pone.0015518

**Published:** 2010-11-29

**Authors:** Joni Macdonald, James D. Glover, Lorna Taylor, Helen M. Sang, Michael J. McGrew

**Affiliations:** The Roslin Institute and Royal Dick School of Veterinary Studies, University of Edinburgh, Roslin, United Kingdom; Brigham and Women's Hospital, United States of America

## Abstract

**Background:**

Avian primordial germ cells (PGCs) have significant potential to be used as a cell-based system for the study and preservation of avian germplasm, and the genetic modification of the avian genome. It was previously reported that PGCs from chicken embryos can be propagated in culture and contribute to the germ cell lineage of host birds.

**Principal Findings:**

We confirm these results by demonstrating that PGCs from a different layer breed of chickens can be propagated for extended periods *in vitro*. We demonstrate that intracellular signalling through PI3K and MEK is necessary for PGC growth. We carried out an initial characterisation of these cells. We find that cultured PGCs contain large lipid vacuoles, are glycogen rich, and express the stem cell marker, SSEA-1. These cells also express the germ cell-specific proteins CVH and CDH. Unexpectedly, using RT-PCR we show that cultured PGCs express the pluripotency genes *c-Myc, cKlf4, cPouV*, *cSox2*, and *cNanog*. Finally, we demonstrate that the cultured PGCs will migrate to and colonise the forming gonad of host embryos. Male PGCs will colonise the female gonad and enter meiosis, but are lost from the gonad during sexual development. In male hosts, cultured PGCs form functional gametes as demonstrated by the generation of viable offspring.

**Conclusions:**

The establishment of *in vitro* cultures of germline competent avian PGCs offers a unique system for the study of early germ cell differentiation and also a comparative system for mammalian germ cell development. Primary PGC lines will form the basis of an alternative technique for the preservation of avian germplasm and will be a valuable tool for transgenic technology, with both research and industrial applications.

## Introduction

Primordial germ cells (PGCs) are the precursors of the germ cell lineage and are restricted to the formation of sperm and eggs in the adult organism. In mammals, PGCs are specified at the beginning of gastrulation. In contrast, in avian species the germ cell lineage is segregated from somatic cell lineages in the epiblast of the laid egg [1]. Early germ cell precursors in chicken embryos can be identified by the expression of the germ cell-specific protein, chicken vasa homologue (CVH) [2]. From a position in the central epiblast, PGCs migrate to an extraembryonic region anterior to the future head region, termed the germinal crescent. From here, at three days of development (stage 15 HH, [3]), the PGCs invade the forming vascular system, congregate in the lateral plate mesoderm conjoining the future gonadal region, and actively populate the developing gonads over the subsequent 48 hours [4]. In the gonad, these primitive germ cells differentiate in accordance with the sexual identity of the surrounding tissues. In the female, germ cells enter meiosis at day 16 of incubation whereas in the male germ cells undergo mitotic arrest and give rise to spermatogonial stem cells which produce functional spermatozoa, beginning at approximately 16 weeks post-hatch.

PGCs in mouse are specified from a region of caudal extra-embryonic mesoderm, much later during embryonic development than in the chicken and can only be propagated for short periods in culture [5]. In specific cell culture conditions, mouse PGCs will ‘de-differentiate’ into cells resembling ES cells, termed EG (embryonic germ) cells [6], [7]. This change in cell fate is thought to occur as mouse PGCs already express several pluripotency markers and respond to growth factors present in the culture medium [8]. A similar de-differentiation process may occur during the formation of germ cell teratomas during embryogenesis [9]. Chicken PGCs can also form EG cells in culture, but it is not known which pluripotency genes are expressed by these cells during this process [10], [11], [12].

It was reported that migratory PGCs could be isolated from the blood of Barred Plymouth Rock layer chickens and expanded in culture for several months [12]. When transplanted to same-sex recipient embryos at stage 13–15 HH, these cells differentiated into functional gametes and generated viable offspring whose genotype derived from the cultured PGCs. Transplantation of the cultured PGCs into opposite-sex recipient embryos did not result in donor-derived functional gametes and the developmental fate of the PGCs in these embryos was not determined.

A robust culture system for chicken PGCs could form the basis of an *in vitro* system for the study of genetic pathways involved in early germ cell proliferation and survival. This will advance our understanding of the mechanisms of early germ cell development and also provide a comparative system which will be informative for studies on mammalian germ cell development. Germline competent PGCs can be developed as a cell-based genetic modification system for the chicken, providing a valuable tool for transgenic technology with both research and industrial applications [13], [14]. This is required as isolated lines of chicken ES (cES) cells do not contribute to the germline after short periods in culture [15], [16], [17]. The only process available for germplasm preservation in poultry is the cryopreservation of semen, which in itself is variable in terms of recovery of functional semen for artificial insemination [18], [19]. Since it is not possible to cryopreserve chicken oocytes and embryos, the development of PGC culture and cryopreservation protocols will provide a means to preserve the germplasm of both males and females and recover the full genetic complement of an avian breed or species.

The key question addressed in this study was whether migratory PGCs could be isolated and cultured from a further breed of chickens and form functional gametes and viable offspring. In addition, we also investigated the intracellular signalling pathways necessary for PGC growth and the pluripotency genes and germ cell-specific markers expressed by cultured PGCs.

## Materials and Methods

### PGC culture conditions

2μl–4μl of blood was isolated from the vasculature system of stage 15–16 HH stage embryos of ISA Brown hens inseminated by ISA brown roosters. Blood was also collected from ISA Brown embryos carrying a single copy lentiviral integrant that contains a transgene that expresses green fluorescent protein (GFP) ubiquitously (Roslin Greens, [20]). Embryos were sexed using primers specific for the W chromosome as described in [21]. Each blood sample was split between two wells of a 48 well tissue culture dish containing 3.0×10^4^ irradiated STO (Sandoz inbred mouse-derived thioguanine-resistant and ouabain-resistant) feeder cells per well and 0.3 ml of PGC culture medium with or without additional growth factors. One third of the culture medium was changed every two days until PGC outgrowth was observed. Thereafter, the total volume of medium was changed every two days. PGC culture medium used was essentially as described in [12] with some modifications. Medium contained 50% BRL (buffalo rat liver) conditioned medium in KO-DMEM (Invitrogen) and contained 10% Fetal Bovine Serum (FBS) (ES cell tested, PAA Laboratories), 2.5% chicken serum (Biosera or Sigma), 2 mM GlutaMax (Invitrogen), 1× NEAA (Invitrogen), 0.1 mM â-mercaptoethanol (Invitrogen), 1× nucleosides (Invitrogen), 1 mM pyruvate (Invitrogen), 1× Penicillin-Streptomycin (Sigma). Growth factors (human bFGF, mouse and human SCF) were obtained from R&D Biosystems. Charaterised FBS (Hyclone) and PAA-Gold FBS (PAA Laboratories) did not support PGC derivation under these conditions, n = 1/151 and n = 0/60, respectively. Inhibitors were obtained from Calbiochem (LY294002 and PD0325901) and prepared according to manufacturer's protocols. Cells were treated with inhibitors (LY294002, 10 µM [22]) (PD0325901, 1 µM [23]) or vehicle every two days.

### Immunohistochemistry and *in situ* hybridisation analysis

PGCs were fixed using 4% paraformaldehyde in PBS for 10 min at room temperature. Primary antibodies were added (rabbit anti-CVH (1∶250), rabbit anti-CDH (1∶250), mouse anti-Tuj III (1∶200, Covance), mouse anti-SSEA1 (1∶40, Developmental Studies Hybridoma Bank)), in 5% goat serum/PBT and samples were incubated overnight at 4°C. Cells were washed for 30 min in PBT and re-incubated with secondary antibodies for one hour (goat anti-rabbit IgG Alexa-Fluor 488, donkey anti-mouse IgG Alexa-Fluor 543, or rabbit anti-mouse IgM Alexa-Fluor 546 for the SSEA1 antibody). Cells were washed for 30 min, counterstained with Hoechst (Sigma), mounted in PBS and imaged directly. The cellular fluorescent stains HCS LipidTOX Green and Mito tracker Red FM CMXRos were used following manufacturer's protocols (Invitrogen). Cells were imaged using an inverted confocal microscope (Nikon eC1; Nikon Instruments). Images were captured using Nikon EZ-C1 Software v3.40.

Whole mount in situ hybridisations were carried out as described [24]. The riboprobe to *cPouV* was described in [25].

### RNA isolation and cDNA synthesis

Total RNA was isolated from cells using RNeasy minikit (Qiagen) according to the manufacturer's guidelines. For cDNA synthesis 1 µg of RNA was heat-treated at 70°C for 10 min and added to the following 20 µl reaction mix: 25 mM MgCl_2_, 4 µl; 10× reverse transcription buffer, 2 µl; 10 mM dNTP mixture, 2 µl; recombinant RNasin, 0.75 µl; random primers, 0.5 µl. Samples were incubated at room temperature for 10 min; 42°C for 55 min; 95°C for five min using the Reverse Transcription System (Promega). For negative controls, the reactions were carried out without reverse transcriptase.

### Reverse Transcription PCR

A 15 µl reaction mixture containing 6 µl H_2_O, 1.5 µl 10× buffer (Roche), 0.3 µl 10 mM dNTPs (Invitrogen), 0.3 µl each primer (50 pmol/µl), 0.1 µl Fast Start Taq (Roche), 3 µl 5× creosol red, and 2 µl sample cDNA. The following reactions were carried out: 95°C for 20 min, followed by 30 cycles of 95°C for 30 sec, annealing temperature for 30 sec, 72°C for one min, and a final extension of 60°C for 30 min. Samples were resolved on a 0.9% TAE agarose gel. Primer sets and annealing temperatures were:

cPouV: TCAATGAGGCAGAGAACACG, TCACACATTTGCGGAAGAAG 58°C

cvh: AGCACAGGTGGTGAACGAACCA, TCCAGGCCTCTTGATGCTACCGA 58°C

c-Myc: GCACAGAGTCCAGCACAGAA, GTTCGCCTCTTGTCGTTCTC 50°C

cKlf4: AGCTCTCATCTCAAGGCACA, GGAAAGATCCACTGCTTCCA 50°C

cSox2: AGGCTATGGGATGATGCAAG, GTAGGTAGGCGATCCGTTCA 50°C cGapdh: CAGATCAGTTTCTATCAGC, TGTGACTTCAATGGTGACA 58°C

cNanog: TTGGAAAAGGTGGAACAAGC, GGTGCTCTGGAAGCTGTAGG 60°C

### Y-irradiation

Fertile eggs (ISA Brown) were irradiated at the laid egg stage prior to incubation using a MDS Nordion Gammacell 1000 Elite with a Cs^137^ source.

### PGC transplantation and host embryo culture

Germline chimeras were generated by injection of GFP^+^ PGCs into the cardiac tract of stage 16 HH embryos. Embryos were transferred into phase III host shells and cultured to hatching as described [26]. The hatched chicks were raised to sexual maturity and genomic DNA samples extracted from semen of adult roosters were screened by semi-quantitative PCR to identify roosters carrying the GFP transgene in the germ cell lineage [27]. Briefly, PCR was carried out on 50 ng of genomic DNA using primers specific for the transgene (CGAGATCCTACAGTTGGCGCCCGAACAG; ACCAGTAGTTAATTTCTGAGACCCTTGTA, annealing temperature: 58°C). In order to estimate the copy number, control PCR reactions were carried out in parallel using 50 ng of non-transgenic DNA spiked with vector plasmid DNA in varying amounts to give the equivalent concentration of one copy per genome (100%), one copy per 10 genomes (10%), one copy per 100 genomes (1%), or one copy per 1000 genomes (0.1%). Founder roosters identified by this method were crossed to stock hens. Offspring were screened for GFP fluorescence to identify birds deriving from the PGCs. All experiments described in this report involving animals, animal breeding, and animal care procedures were reviewed and approved by The Roslin Institute's animal ethics committee. These experiments were performed under specific license from the U.K. Home Office.

### Culture of chicken ES cells

Chicken embryonic stem (cES) cells were isolated and cultured as described in [28] with some changes. The epiblast of GFP^+^ laid eggs was isolated, dissociated, and cultured on either STO or BRL feeder cells in PGC medium containing 80% BRL conditioned medium and 5 ng/ml bFGF. Chicken ES cells were expanded for four to six weeks before mRNA was isolated from two independent lines as described above for the PCR analysis. The two lines of cES cells were further tested for pluripotency after an additional four to six weeks in culture by injection into the sub-germinal cavity of newly laid eggs that were first irradiated at 5.0 Gray (Gy). cES were dissociated from a 24 well plate using cell dissociation buffer (Invitrogen). Cells were resuspended in KO-DMEM and 1 ul of solution (∼500–1000 cells) was injected into the sub-germinal cavity. Injected eggs were transferred to phase II host shells [26] and incubated in these shells for eight days without transfer to new host shells. An embryo containing GFP^+^ cells from each line was cryosectioned to assay for GFP^+^ cell contribution to host tissues.

### Statistical analysis of inhibitor experimental data

For PGC culture derivation the no added growth factors condition was compared individually to each of the other culture conditions and the data statistically validated using a Paired Student T-Test with two tailed distribution. A Paired Student T-Test with two tailed distribution was also used to compare the data from the inhibitor experiments where vehicle was compared with experimental.

## Results

### Propagation of PGCs *in vitro*

**Figure 1:**
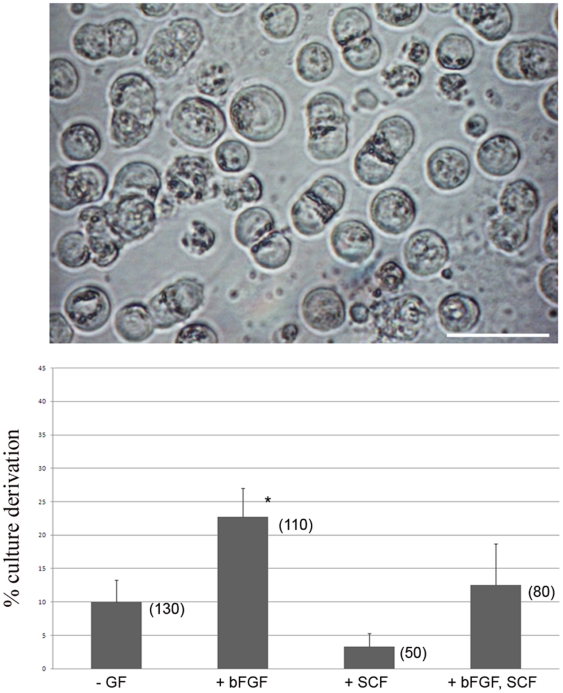
*In vitro* culture of PGCs. **Top:** PGCs from a representative culture were imaged using brightfield microscopy. Doublets indicative of dividing cells are visible in the culture. Bar, 50 µm. **Bottom:** Blood from single embryos was split into two wells and cultured with or without additional growth factors. Wells containing more than 100 non-adherent PGCs at three weeks were scored as positive. Cultures contained no additional growth factors or 2.5 ng/ml bFGF with or without 5 ng/ml SCF. (n) indicates the number of cultures assayed. Error bars, S.E.M. *, p<0.05.

Long term *in vitro* culture of PGCs and germline transmission has been demonstrated for PGCs deriving from Barred Plymouth Rock chickens [12]. We attempted to repeat and extend this investigation using a different breed of layer-type chickens, the ISA Brown. Embryonic blood containing migratory PGCs was isolated from day 3 (Stage 16 HH) embryos and cultured on a layer of STO feeder cells. Culture medium contained both chicken and fetal bovine animal sera, conditioned medium from BRL cells, bFGF and SCF (see Materials and Methods). After two weeks in culture PGCs were present in the majority of culture wells and by three weeks blood cells in the wells had lysed. The cells remaining in several wells per experiment displayed the described morphology of PGCs (Fig. 1) [12], [29].

We carried out a large number of experiments in parallel, to determine which commercially-available FBS and chicken sera supported PGC survival and which growth factors were required as additives to the basic medium. We defined a successful culture derivation as more than 100 PGCs being present in the culture at the end of three weeks. Several sources of fetal bovine and chicken sera did not support the growth of PGCs (see Materials and Methods). Using selected serum conditions (Materials and Methods) we assayed if the addition of bFGF and SCF improved the frequency of PGC culture derivation (n = 370) (Fig. 1). We found that addition of bFGF significantly increased PGC culture derivation but addition of SCF did not. Several lines of cultured PGCs were expanded from single embryo blood samples (seven lines, cell number >100,000 for each) and used for the subsequent experiments. PCR analysis of these lines for a female-specific W chromosome [21] revealed that all lines isolated were male.

### Propagation of PGCs is dependent on PI3K and MEK signalling

**Figure 2:**
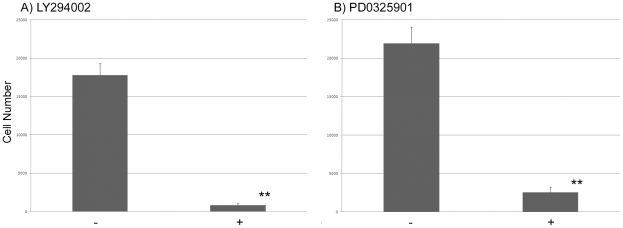
PI3K and MEK are necessary for PGC proliferation. PGCs (1000) were seeded into a well and grown in the presence of pharmalogical inhibitors or vehicle for seven days in medium containing 2.5 ng/ml bFGF and total cell number was assayed. **A**) LY294002, (10 µM). **B**) PD0325901, (1 µM). Three lines of cPGCs were assayed between 3–6 times in three separate experiments. Error bars, S.E.M. **, p<0.01.

We assayed the effect of inhibiting phosphatidylinositol-3kinase (PI3K) on PGC propagation using the inhibitor, LY294002. PI3K is activated by many signalling pathways, including the c-kit receptor [30]. The c-kit ligand, SCF, is a known survival factor/mitogen for mouse primordial germ cells [31], [32], [33]. PGCs were grown in PGC culture medium containing inhibitor dissolved in vehicle or vehicle alone and cell number was assayed after one week. We observed that PGC proliferation was severely inhibited in the presence of LY294002 (Figure 2A). Cells were assayed for viability by the cellular exclusion of trypan blue. Most cells in the inhibitor treated wells were trypan blue positive (90% inhibitor treated, <10% vehicle treated cells) after seven days indicating that cell death was increased in the presence of inhibitor.

We next assayed if the FGF/MAP kinase pathway was necessary for PGC proliferation by treating cultured PGCs with PD0325901, a potent inhibitor of MEK [34]. FGF has been shown to be a survival factor and activate MAP kinase in mouse migratory PGCs [35]. PGCs were again grown in medium containing inhibitor dissolved in vehicle or vehicle alone and cell number was assayed after one week. PGC number was significantly reduced in the presence of the MEK inhibitor (Fig. 2B). A trypan blue cellular exclusion assay revealed that cell death increased in the presence of the inhibitor (90% inhibitor treated, <10% vehicle treated cells) after seven days in culture. These results demonstrate that signalling through PI3K and MEK are necessary for PGC growth in culture.

### Characterisation of cultured PGCs

**Figure 3:**
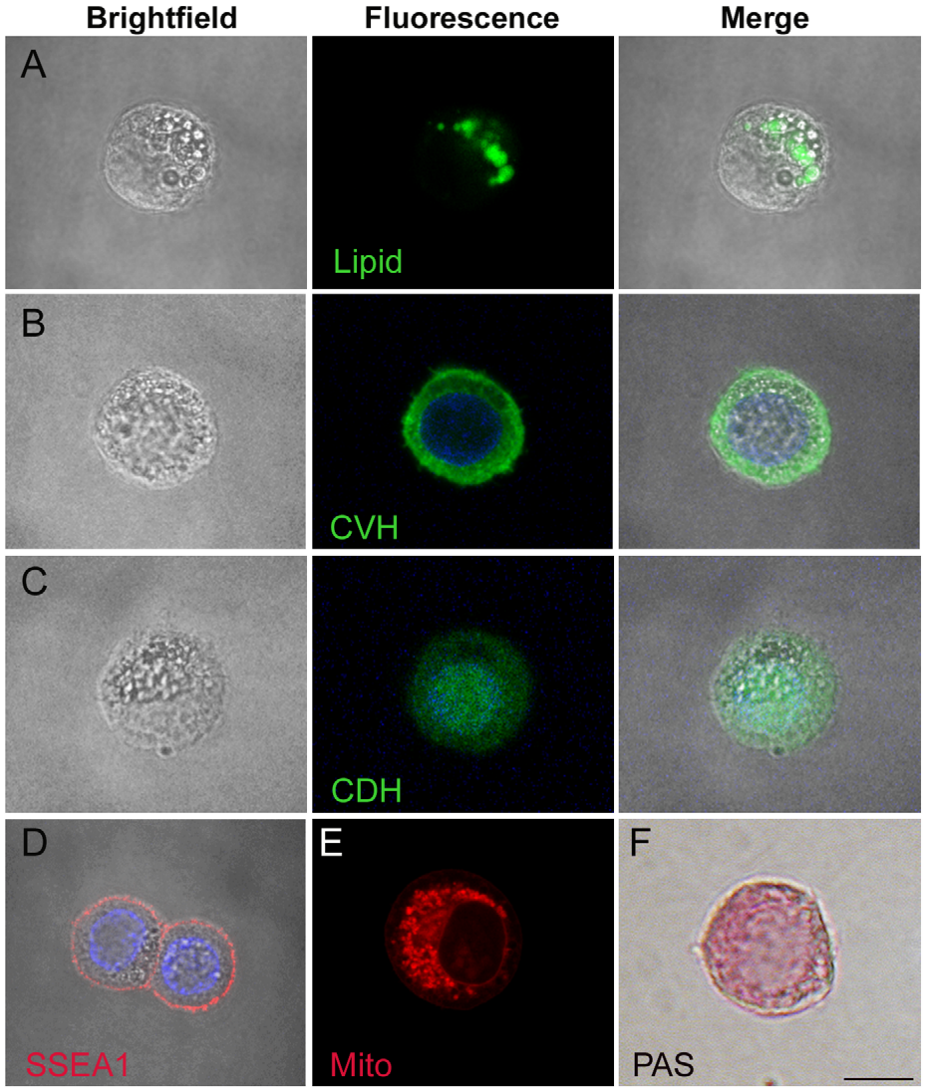
Sub-cellular localisation of germ cell markers in PGCs. Immunofluorescence of select germ cell markers was carried out on two separate lines of PGCs. Staining patterns for both lines were equivalent. **A**) LipoTox, a marker of neutral lipid. **B**) CVH, chicken vasa homologue. **C**) CDH, chicken dead end homologue. **D**) SSEA-1. **E**) Mito Tracker Red. **F**) PAS staining. Bar, 10μm.

To examine the cellular morphology of cultured PGCs and the intracellular localisation of germ cell-specific proteins, we carried out immunofluorescence on two PGC lines maintained *in vitro* for three months and 12 months. The cultured PGCs contain a large nucleus and many prominent vacuoles (Fig. 3). To determine the contents of the vacuoles we stained the PGCs with LipoTox, a marker of neutral lipids. This revealed that many of the larger vacuoles contain neutral lipid (Fig. 3A). We also carried out the classic Periodic acid-Schiff (PAS) reaction on the PGCs, a stain for cellular glycogen. PAS staining produced a diffuse staining pattern throughout the cytoplasm indicating a cytoplasm rich in glycogen particles (Fig. 3F). Staining with Mitotracker Red, an active mitochondrial marker also revealed dispersed functional mitochondrial throughout the cytoplasm (Fig. 3E). Immunostaining with the ES cell marker, SSEA-1 demonstrated that the cell surface of PGCs stained strongly for this epitope (Fig. 3D).

To determine if the cultured PGCs continued to express the germ cell-specific proteins found in migratory PGCs *in ovo*, we used immunofluorescence to detect CVH, chicken vasa homologue, and CDH, chicken dead end homologue; two RNA processing proteins important for germ cell survival and specification [2], [36], [37], [38], [39]. Immunostaining with an antibody to CVH illustrated that in most cells CVH was localised throughout the cytoplasm (Fig. 3B). This is consistent with the reported cytoplasmic localisation of CVH in avian germ cells [2]. Immunostaining with CDH antibody displayed a strong nuclear localisation and diffuse staining throughout the cytoplasm (Fig. 3C). This result is consistent with the reported description of CDH as a nuclear-localised protein in migratory and post-migratory PGCs [39]. We conclude from these results that the expression of these germ cell-specific proteins is maintained in cultured PGCs.

### Cultured PGCs express a set of pluripotency genes

**Figure 4:**
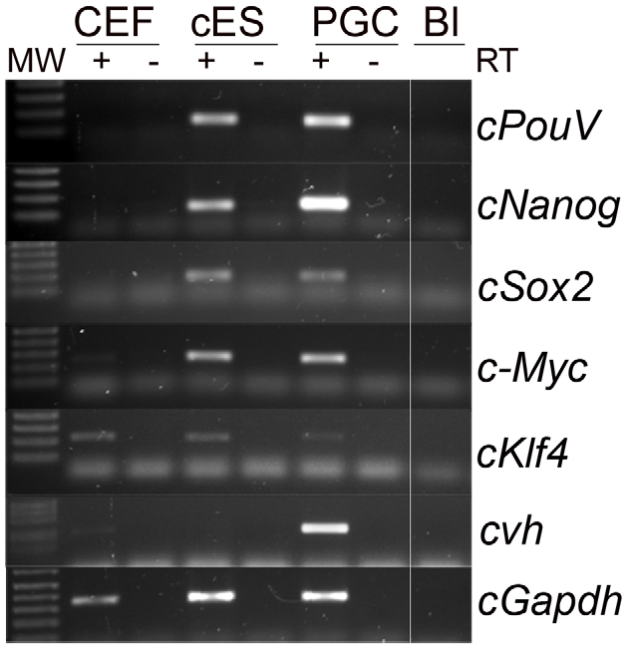
PGCs express many pluripotency genes. RT-PCR was carried out on cDNA samples from two independent lines of PGCs and cES cells. CEF, chicken embryonic fibroblasts; Bl, blank-no cDNA control.

We subsequently examined the expression of the known pluripotency markers *cPouV*, *cSox2*, *cNanog*, *cKlf-4*, and *c-Myc* in cultured PGCs. During germ cell specification in the mouse, nascent germ cells begin to express *Oct3/4*, *Nanog*, and *Sox2*, and express *c-Myc* and *Klf4* only upon conversion to embryonic germ (EG) cells [40], [41]. We examined the expression of the chicken homologues of these four genes in cultured PGCs, cES cells, and chicken embryonic fibroblasts (CEFs). The cES cells used in this study were shown to contribute to the three germ layers of the forming chicken embryo in chimeras (Fig. S1). We isolated RNA from CEFs, STO feeder cells, cES cells and cultured PGCs and carried out RT-PCR analysis (Fig. 4). The germ cell-specific marker *cvh* was used as a positive control for PGC-specific gene expression and was found to be expressed in cultured PGCs and not in cES cells. We found that cES cells expressed all four of the pluripotency markers, *cPouV*, *cSox2*, *cKlf-4*, and *c-Myc*, and also *cNanog*. Surprisingly, we observed that cultured PGCs also expressed all of these pluripotency genes (Fig. 4). STO feeder cells did not express any of these chicken genes (data not shown). CEFs expressed *cKlf-4* and a low level of *c-Myc*. *Klf4* and *c-myc* are expressed in many tissues during embryogenesis in mouse and rat and are not strictly markers of pluripotency alone [42], [43], [44]. These data show that the PGCs express many pluripotency genes in common with cES cells.

### Cultured PGCs colonise the forming gonad and undergo meiosis

**Figure 5:**
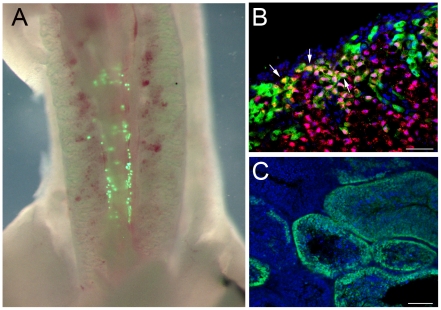
PGCs colonise the gonad and undergo meiosis in temporal accordance with the host embryo. **A**) Ventral view of a day 5 chicken embryo that was injected at stage 16 HH with GFP^+^ cultured PGCs. The GFP^+^ cells are found near the forming genital ridges. **B**) Section of ovary from a Day 7 hatchling immunostained for Scp3. Some GFP^+^ PGCs are positive for the meiotic marker, arrows. Bar, 50ìm **C**) Section of a seminiferous tubule for 16 week old male host. GFP+ cells are present and juxtaposed to the basement membrane. Scp3, red; Blue, nuclear stain. Bar, 100μm.

To validate that cultured PGCs formed functional germ cells, i.e. colonise the forming gonad and differentiate into functional gametes, we first tested the cells for their ability to migrate to the gonad. We used cultured lines of male PGCs that had been generated from a transgenic line of chickens that expressed GFP ubiquitously (GFP^+^, [20]). GFP^+^ PGCs were injected into the vascular system of day 3 embryos *in ovo* (stage 16 HH). Within two hours of injection, GFP^+^ cells were clustered in the lateral plate mesoderm in the caudal region of the embryo (data not shown). Embryos were resealed and incubated until day 5 of development (stage 26HH). An examination of the ventral aspect of the embryo revealed that GFP^+^ cells were clustered along the ventral midline of the embryo surrounding the forming genital ridges (Fig. 5A). By day 10 of development, GFP^+^ cells could be seen throughout the developing gonad (n = 3 of 3, data not shown).

We extended this analysis by examining the gonads of sexually mature (16 weeks post hatch) recipient roosters. In the gonads of these birds, GFP^+^ cells were located adjacent to the basement membrane in the seminiferous tubules (Fig. 5C, n = 3 of 3). A region of GFP^+^ cells extended from the basement membrane partially toward the luminal surface of the tubule. The GFP fluorescence from the lentiviral transgene is not detectable in mature spermatids [20] so we could not determine by immunohistochemistry if the donor PGCs were forming functional spermatozoa. We next examined the fate of the male PGCs in female gonads. Sections from ovaries from recipient female hatchlings were examined for the presence of GFP^+^ cells. GFP^+^ cells were located in the cortex of the ovaries of these birds (Fig. 5B). We examined the expression of the meiotic marker, Scp3, to determine if the injected PGCs could undergo sex-specific differentiation in females (Fig. 5B). We observed that many of the GFP^+^ cells in the ovarian cortex co-expressed Scp3 indicating that these cells were entering meiosis in accordance with the host embryo (n = 3 of 3). Thus, the cultured male PGCs were able to colonise both male and female gonads and differentiate.

### Germline transmission of cultured PGCs

**Figure 6:**
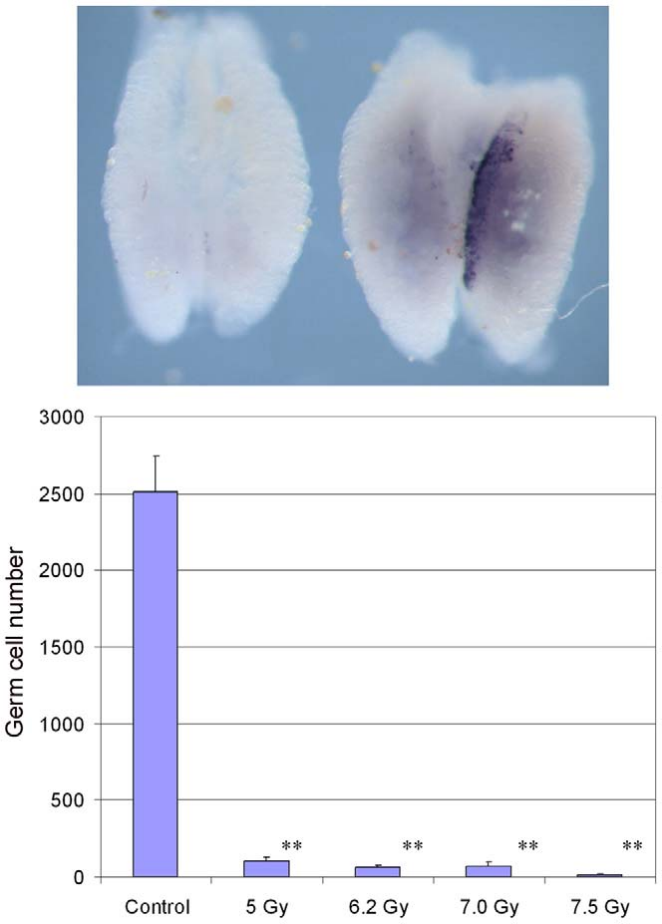
Y-irradiation of host embryos ablates endogenous PGCs. **Top:** *In situ* hybridisation with a riboprobe to *cPouV* of day 5 gonads from a control and 7.5 Gy irradiated embryo. **Bottom:** Germ cell numbers in control embryos and embryos irradiated for various times. Error bars, S.E.M. **, p<0.01.

We next tested if the cultured PGCs were germline competent, i.e. would these cells form functional gametes and produce GFP^+^ hatchlings when recipient birds were mated to wildtype birds. To increase the contribution of the donor PGCs to the host gonad we first determined if ã-irradiation would deplete the recipient embryo of endogenous PGCs. Fertile laid eggs were irradiated at selected doses of ã-irradiation, from 5–7.5 Gy, and incubated for six days. We observed that at doses above 5 Gy, embryonic development was delayed by 24 hours such that six day incubated embryos exhibited the morphological development of day 5 (stage 26HH) embryos. We carried out *in situ* hybridisation analysis using a riboprobe for *cPouV* to visualise the germ cells in the embryo (Fig. 6, top). Embryos were sectioned and *cPouV* expressing cells were counted. At doses above 5.0 Gy, germ cell number was significantly reduced (Fig. 6, bottom): at 5.0 Gy, the average germ cell number in day 5 embryos was 101. 4±23.6, at 7.0 Gy, PGC number was 70.2±27.1, at 7.5 Gy, PGC number was 13.3±4.1. Control day 5 embryos contained 2508±235 PGCs. We found that the highest dose of 7.5 Gy compromised development (50% survived to day 16 versus 64% for 7.0 Gy), so we used the lower dose of 7.0 Gy for recipient embryos.

To demonstrate that the cultured PGCs were germline competent, we injected the cells into host embryos and raised these birds to sexual maturity. We injected GFP^+^ PGCs isolated from a single GFP^+^ transgenic embryo (10-08-09) in FGF supplemented medium and which had been propagated for 53 days in culture. 100–500 PGCs were injected into day 3 (stage 16 HH) embryos *in ovo* that were either non-irradiated or irradiated at 7.0 Gy. Embryos were incubated until hatch. The results from two separate experiments are shown in Table 1. 26 embryos were injected and 12 embryos survived to hatch (7 males, 5 females). Of these embryos, 71% of non-irradiated (5/7) and 37% of irradiated embryos (7/19) survived. The hatchlings were raised to sexual maturity and semen samples were assayed from the roosters for the presence of the GFP transgene. All roosters contained DNA deriving from the donor PGCs in their semen at estimated frequencies from 1–30% (see materials and methods). Three roosters containing the highest estimated contribution from donor PGCs in their semen were crossed with wildtype hens and the hatched chicks were screened for GFP fluorescence. As shown in Table 1, germline transmission of the injected PGCs was observed with all three roosters. The corrected frequency of transmission was between 2–16% which correlated well with the estimated frequency of transgenic DNA in the semen samples. The number of roosters screened was too small to confirm that prior irradiation of host embryos increased the contribution of donor PGCs to the germline, although the results indicate that this may be correct. A post mortem examination of the testes (n = 7 of 7) showed that GFP^+^ cells were present in all the birds. The amount of fluorescent tissue correlated well with PCR expression data.

**Table 1:** Frequency of germline transmission of donor GFP+ cPGCs in host roosters.

Founder Birds♂	Eggs set	Chicks Screened (%)	% genome equivalents in semen	GFP^+^offspring (% transmission*)
**PGC 2–13**	242	147 (61%)	6	2 (2.8%)
**PGC 3–6**	242	83 (34%)	30	7 (16.8%)
PGC 3–11	190	110 (58%)	2	1 (1.8%)
PGC 2–3	-	-	1	-
PGC 3–5	-	-	4	-
PGC 3–10	-	-	1	-
PGC 3–12	-	-	1	-
Founder Birds♀	Embryos examined	GFP^+^ embryos		
**PGC 2–2**	63	0		
**PGC 2–7**	57	0		
**PGC 3–3**	68	0		

Irradiated birds are shown in bold.

*the actual transmission rate is double the observed number of GFP^+^ chicks due to the heterozygosity of the GFP allele and meiotic reduction.

Three recipient hens that were injected with GFP^+^ PGCs (Table 1) were crossed with wild type males and embryos from these crosses were screened for GFP fluorescence. No GFP fluorescence was observed in these embryos (n = 0 of 188). The recipient hens were sacrificed and their ovaries were examined for the presence of GFP^+^ cells. No GFP fluorescence was observed in the ovaries of these birds (shown = 3 of 3)). Since GFP^+^ cells had been present in cortex of female birds examined at hatch (Fig. 5B, above), we conclude that the donor male PGCs were lost from the female ovary during sexual maturation. These results indicate that the donor male PGCs form functional gametes in male recipients but suggest that they are lost from the ovaries of female recipients during oogenesis.

## Discussion

### Characterisation of chicken PGCs

We have shown that cultured PGCs have the expected distinctive cellular morphology consisting of a large nucleus and a cluster of large vacuoles present in the cytoplasm (Fig. 3). Using a lipid stain, we demonstrated that lipid is present within some of the larger vacuoles of the cultured PGCs. This is not simply an *in vitro* culture artefact as chicken circulatory PGCs *in ovo* were also reported to contain large lipid vacuoles [29]. The lipid rich cytoplasm observed in chicken PGCs is similar to that of migratory human PGCs [45], [46] but is unlike the cytoplasm of migratory PGCs in both the mouse and the pig which do not contain lipid vacuoles [47], [48]. The high glycogen content and diffuse staining seen in cultured PGCs agrees with previous observations in which PAS staining was also used to identify migratory chicken PGCs [11], [29], [49]. Active mitochondria were found to be present throughout the cytoplasm of cultured PGCs. The cell surface marker, SSEA-1, has been shown to be present on various undifferentiated progenitor cells including ES cells and EG cells [8]. Here, SSEA-1 was also shown to be present on cultured PGCs. This is in line with previous descriptions of SSEA-1 expression on both chicken and mouse PGCs [49], [50], [51].

The germ cell-specific protein CVH was restricted to the cytoplasm of cultured PGCs. In Drosophila, VASA is a member of a DEAD-box RNA helicase family specific to germ cells [36]. It is indispensible for development through regulation of mRNAs such as *Nanos*. The mouse vasa homologue (MVH) was discovered to play a role in RNA processing of both mRNAs and piRNAs in germ cells and to be localised to cytoplasmic granules some of which are closely associated with mitochondria [38], [52]. It will be of interest to determine if CVH displays a similarly sub-cellular localisation in cytoplasm of chicken PGCs.

The germ cell protein dead end functions to neutralize the inhibitory effects of several miRNAs allowing the expression of key genes, such as *Nanos* in PGCs [53]. In mouse *dead end homologue* mutants, a loss of PGCs and an increased susceptibility to spontaneous testicular germ cell tumour formation was observed [37]. In this work we observed that some CDH was present in the cytoplasm of cultured PGCs. This is in contrast to previous observations where CDH was described as exclusively nuclear in chicken PGCs [39]. It has been shown in zebrafish PGCs that dead end is localised predominantly to perinuclear granuoles in the cytoplasm and is thought to play a role in shuttling mRNAs between the nucleus and cytoplasm [54]. It is possible that CDH could be acting in a similar manner in chicken PGCs.

### Expression of pluripotency genes in chicken PGCs

Mouse PGCs can be propagated *in vitro* for short periods before undergoing apoptosis [55]. In the presence of bFGF, SCF, or high levels of activation of the AKT signalling pathway that is downstream of the SCF receptor, mouse PGCs will de-differentiate into EG cells [5], [56]. Mouse PGCs express the pluripotency markers, *Oct3/4*, *Nanog, Klf2*, and *Sox2*. Upon de-differentiation into EG cells, *c-Myc* and *Klf4* expression is initiated, suggesting that the expression of these additional factors may be sufficient to achieve a pluripotent state [41]. Similarly, it has been shown that porcine neonatal spermatogonial stem cells (SSCs) express low levels of *Oct3/4, c-Myc*, and *Sox2* and these levels and those of *Nanog* and *Klf4* increased during culture concomitant with the acquisition of pluripotency [57]. Indeed, the over-expression of c-Myc, Sox2, Klf4, and Oct3/4 transcription factors are sufficient to reprogram a somatic cell to pluripotency [58].

Here, we have shown that cultured chicken PGCs express *c-Myc*, *cSox2*, *cKlf4*, *cPouV* and *cNanog*, similar to the expression pattern seen in pluripotent cES cells (Fig. 4 and [25]). This suggests that the lineage restriction of chicken germ cells to gamete formation may not be due to the absence of pluripotency factors but may lie in epigenetic modifications of the genome or by the action of germ cell-specific proteins including CDH and CVH. It will be of interest to determine whether the expression of homologues of the pluripotency genes in other vertebrate species with early segregation of the germ cell lineages such as *Xenopus*, medaka, and zebrafish is comparable to chickens.

### The *in vitro* propagation of chicken PGCs

We found that the addition of bFGF to culture medium increased the frequency of derivation of PGC cultures (Fig. 1). However, the addition of SCF, with or without bFGF, did not increase the frequency of PGC culture derivations. Although we had predicted that addition of SCF would increase the isolation of PGC cultures, as SCF is a know survival factor for mouse PGCs [31], [32], [33], our observations may reflect the levels of SCF in the culture media tested. BRL cells are known to secrete the growth factors LIF, SCF, and IGF-1 [59], [60], [61], so additional SCF may not be needed in the presence of BRL conditioned medium. As noted above, increased SCF/c-kit signalling can drive the conversion of PGCs into EG cells and inhibit PGC propagation [5], [56].

We also observed that both the MEK inhibitor, PD0325901, and the PI3K inhibitor, LY294002, significantly inhibited growth of PGCs in culture. Similar results were seen for inhibition of MEK/MAPK signalling in mouse PGCs [22], [35]. However, it was also reported that inhibition of PI3K signalling in mouse PGCs, using the equivalent concentration of inhibitor, had no effect on germ cell numbers [22]. This difference in experimental results could be attributed to the increased proliferation of chicken PGCs under our culture conditions in comparison to mouse PGC cultures. In our inhibitor assays, which were initiated with approximately 1000 PGCs per well, we saw a ∼20-fold increase in control cell number in seven days, whereas in the mouse PGC culture experiments, control PGC number increased only 2-fold in seven days [22]. This increase in cell number could make more subtle changes in cell proliferation apparent.

These results suggest that signalling through both PI3K pathway and MAP kinase pathways are necessary for chicken PGC proliferation in culture. SCF/c-kit signalling through PI3K has also been shown to activate MAPK in haematopoietic progenitor cells [62]. Furthermore, signalling through the FGF receptor has been shown to activate PI3K in some cell types [63]. Therefore, a more detailed examination of these pathways will be needed to ascertain the specific receptor-mediated transduction pathways functioning in PGCs. An in depth understanding of the factors required for PGC proliferation in culture will form the basis of developing a defined, serum-free culture medium for chicken PGCs.

### Sex-specific differences in chicken PGCs

Mouse PGCs in opposite-sex recipients have been shown to enter meiosis and differentiation in accordance with the developmental age of the recipient embryo [64]. Male PGCs in female mice will form functional oocytes [65]. Female PGCs cannot form functional gametes in the male gonad but they do undergo the initial steps of spermatogenesis in the male gonad [66]. In the chicken, cultured PGCs did not form functional gametes when transplanted into opposite-sex recipient embryos [12]. Our data support this finding. We have demonstrated that male PGCs transplanted to female recipients have entered meiosis by hatch. Subsequently, these cells may be lost from the female ovary. These results are similar to those obtained when primary male migratory PGCs (day 3, stage 14–15) were transplanted into female recipients; <1.0% of offspring of opposite-sex germline chimeras were descended from the donor PGCs [67]. Our results suggest that, in contrast to mammalian germ cell development, male chicken PGCs do enter meiosis in developmental accordance with the host ovary and may be lost during post-natal development, indicating sex-specific differences in chicken germ cells.

The work presented here confirms the long-term propagation of primordial germ cells from chicken embryos and their competence to contribute to the germline of recipient birds. These cells will be a valuable tool for transgenic technology with both research and industrial applications and will be useful to study the genetic pathways involved in germ cell proliferation, migration, and determination.

## Supporting Information

Figure S1 **Chicken ES cells contribute to the three germ layers of the developing embryo.** **A**) Day 8 embryo that was injected with GFP^+^ cES cells at the laid egg stage. **B**) GFP^+^ cES cells after three weeks in culture. **C**) Transverse section of the neural tube showing GFP^+^ neurons. **D**) Longitudinal section of the forming limb. GFP^+^ cells are in the mesoderm surrounding the forming nerve tracts **E**) Transverse section of the intestine at the level of the liver demonstrating GFP^+^ cell contribution to the endodermal cell layer. Nuclear stain, blue; Tuj III neuronal marker, red. Bar, 200m.
(TIF)
