# Supplementary figures and images for: Characterisation and Germline Transmission of Cultured Avian Primordial Germ Cells
Source: PLoS One. 2010 Nov 29;5(11):e15518. doi: 10.1371/journal.pone.0015518 (PMC2993963; doi:10.1371/journal.pone.0015518)

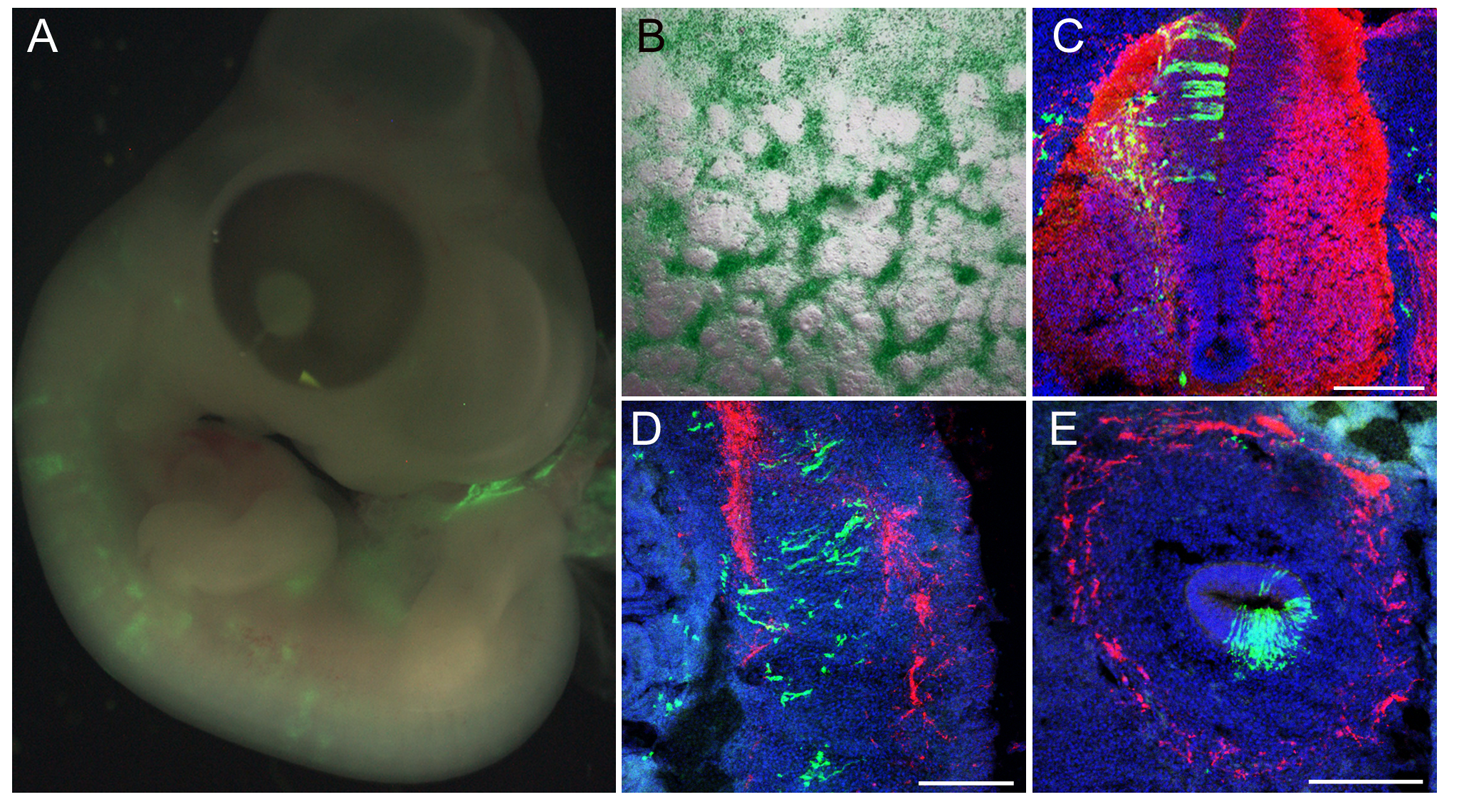

Supplement: Figure S1 — Chicken ES cells contribute to the three germ layers of the developing embryo. A) Day 8 embryo that was injected with GFP+ cES cells at the laid egg stage. B) GFP+ cES cells after three weeks in culture. C) Transverse section of the neural tube showing GFP+ neurons. D) Longitudinal section of the forming limb. GFP+ cells are in the mesoderm surrounding the forming nerve tracts E) Transverse section of the intestine at the level of the liver demonstrating GFP+ cell contribution to the endodermal cell layer. Nuclear stain, blue; Tuj III neuronal marker, red. Bar, 200m. (TIF) [file pone.0015518.s001.tif]
